# Enhanced CO_2_/CH_4_ Separation Performance of a Mixed Matrix Membrane Based on Tailored MOF‐Polymer Formulations

**DOI:** 10.1002/advs.201800982

**Published:** 2018-08-02

**Authors:** Yang Liu, Gongping Liu, Chen Zhang, Wulin Qiu, Shouliang Yi, Valeriya Chernikova, Zhijie Chen, Youssef Belmabkhout, Osama Shekhah, Mohamed Eddaoudi, William Koros

**Affiliations:** ^1^ School of Chemical and Biomolecular Engineering Georgia Institute of Technology 311 Ferst Drive Atlanta GA 30332‐0100 USA; ^2^ Advanced Membranes and Porous Materials Center Division of Physical Science and Engineering King Abdullah University of Science and Technology Thuwal 23955 KSA

**Keywords:** mixed‐matrix membranes, metal organic frameworks, natural gas separation, subambient conditions

## Abstract

Membrane‐based separations offer great potential for more sustainable and economical natural gas upgrading. Systematic studies of CO_2_/CH_4_ separation over a wide range of temperatures from 65 °C (338 K) to as low as −40 °C (233 K) reveals a favorable separation mechanism toward CO_2_ by incorporating Y‐*fum*‐**fcu**‐MOF as a filler in a 6FDA‐DAM polyimide membrane. Notably, the decrease of the temperature from 308 K down to 233 K affords an extremely high CO_2_/CH_4_ selectivity (≈130) for the hybrid Y‐*fum*‐**fcu**‐MOF/6FDA‐DAM membrane, about four‐fold enhancement, with an associated CO_2_ permeability above 1000 barrers. At subambient temperatures, the pronounced CO_2_/CH_4_ diffusion selectivity dominates the high permeation selectivity, and the enhanced CO_2_ solubility promotes high CO_2_ permeability. The differences in adsorption enthalpy and activation enthalpy for diffusion between CO_2_ and CH_4_ produce the observed favorable CO_2_ permeation versus CH_4_. Insights into opportunities for using mixed‐matrix membrane‐based natural gas separations at extreme conditions are provided.

Natural gas consisting primarily of methane (CH_4_) is an important commodity and chemical feedstock with a relatively low CO_2_ footprint.^1^ Nevertheless, raw natural gas contains various impurities, and a key step in natural gas treatment is the removal of acid gases, e.g., CO_2_, to prevent corrosion of the pipelines.^2^ Conventional amine absorption, a mature technology for CO_2_ removal with high selectivity, suffers from a number of well‐documented drawbacks.^3^ Nowadays, membrane‐based technology is recognized as a highly promising and energy‐efficient alternative to amine‐based technology, as it offers flexible design, smaller footprint, and is relatively eco‐friendly.[[qv: 2b,3]] However, present organic polymer membranes face a limit of trade‐off between permeability and selectivity,^4^ while inorganic membranes face scalability and cost limitations.^5^ Hybrid membranes, also called mixed matrix membranes (MMMs), combining a dispersed molecular sieving phase (filler particles) and a continuous polymer (matrix), offer opportunities to overcome the drawbacks associated with pure polymer‐ or inorganic‐based membranes. Various dispersed fillers, such as zeolites,^6^ mesoporous silicas,^7^ activated carbons,^8^ carbon nanotubes,^9^ and metal organic frameworks (MOFs),^10^ have been incorporated into polymers to produce MMMs. Such MMMs must display compatibility between the dispersed and continuous constituents.^11^ In this regard, MOFs are especially attractive in terms of compatibility with polymers due to their integrated organic moieties in organic linkers.^11^, ^12^ Nevertheless, relatively few reported MOF‐based MMMs (MOF‐based MMMs) exhibit enhanced CO_2_ permeability and CO_2_/CH_4_ selectivity as compared to the associated parent pure polymer membrane.^13^

The ability to increase the loading ratio of MOFs to polymers offers the opportunities to improve the separation performance of MOF MMMs if aggregation of MOF particles at high loadings in the polymers matrix can be controlled and avoided.[[qv: 5b]] The choice of the matrix polymer with the appropriate and intrinsic permeability are vital for the successful integration of the filler properties in the MMM. Kulkarni and co‐workers developed a novel hollow fiber membrane process at subambient temperatures offering an enhancement in the CO_2_/N_2_ selectivity for custom‐synthesized pure polyimide membranes by twofold to fourfold at temperatures below −20 °C with negligible CO_2_ permeance loss as compared to ambient temperature values.^14^ It is important to note that feed streams in natural gas separation processes can be heated to 65 °C or higher (to prevent condensation in membrane modules) and cooling pretreatment can be used to capture and remove valuable ethane and higher hydrocarbon components. On this basis, evaluating the natural gas separation at extreme temperatures, beyond typical temperatures (25–35 °C) is of practical importance.

This communication reports a novel approach to achieve exceptionally high CO_2_/CH_4_ separation performance for preselected MOF‐based MMMs without altering the composition of the integrated MOF or polymer. Principally, we report the high performance of the MOF‐based MMM derived from Y‐*fum*‐**fcu**‐MOF with interconnected tetrahedral (5.2 Å) and octahedral (7.6 Å) cages (**Figure** **1**a). The engineered **fcu**‐MOFs have triangular windows as the sole entrance for guest molecules to the MOF pore system.^15^ Our previous work had demonstrated the good CO_2_/CH_4_ separation performance of Y‐*fum*‐**fcu**‐MOF incorporated 6FDA‐DAM polyimide membrane at 35 °C (308 K).^16^ In this work, we extend our investigation to show that controlling the temperature is a promising way to further fine‐tune the performance attributes of hybrid membranes.

**Figure 1:**
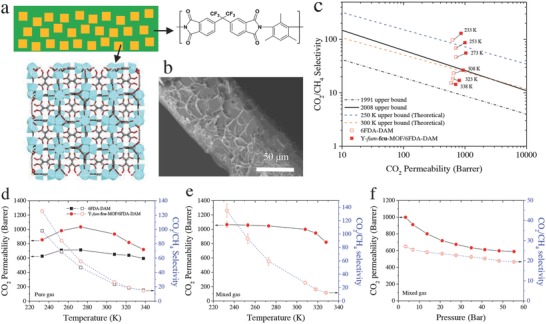
a) Illustrative schematic showing the principal of fabricating the hybrid membrane by incorporating 20% Y‐fum‐**fcu**‐MOF crystals into 6FDA‐DAM polymer. b) SEM images of the fabricated 20% Y‐fum‐**fcu**‐MOF/6FDA‐DAM membrane. c) The effect of temperature on single gas CO_2_/CH_4_ separation performance of the membranes in comparison with upper bounds. d) Variation of CO_2_ permeability and CO_2_/CH_4_ ideal selectivity as a function of temperature. All the single gas tests were performed with CO_2_ pressure of 1.38 bar and CH_4_ pressure of 4.14 bar. CO_2_/CH_4_ (50/50) mixed gas tests for 20% Y‐fum‐**fcu**‐MOF/6FDA‐DAM membrane with e) temperature variation at 3.5 bar and f) pressure variation at 308 K.

Single gas permeation tests for pure CO_2_ (at 1.38 bar) and CH_4_ (at 4.14 bar) were performed on the Y‐fum‐**fcu**‐MOF/6FDA‐DAM membrane with 20 wt% MOF loading (Figure 1b) and pure 6FDA‐DAM membrane at various temperatures ranging from 233 to 338 K, as shown in Figure 1c. As expected, decreasing the temperature significantly affects the CO_2_/CH_4_ separation performance, especially selectivity, for both pure 6FDA‐DAM membrane and hybrid Y‐fum‐**fcu**‐MOF/6FDA‐DAM membranes. The CO_2_/CH_4_ permselectivity dramatically increases with decreasing temperature, and interestingly, the CO_2_ permeability does not change much, resulting in a notable enhancement of the membrane performance exceeding the upper‐bounds reported by Robeson.^17^ Comparing with the parent 6FDA‐DAM membrane, the hybrid Y‐fum‐**fcu**‐MOF/6FDA‐DAM membrane exhibits a higher CO_2_/CH_4_ separation performance especially at subambient temperatures, such as 253 and 233 K, surpassing the pure polymer upper bounds pertaining to temperature effects.^18^ At 233 K, the hybrid Y‐fum‐**fcu**‐MOF/6FDA‐DAM membrane exhibits an exceptionally high CO_2_/CH_4_ selectivity (≈126) associated with a high CO_2_ permeability (≈855 barrers), reflecting approximately a fourfold enhancement in CO_2_/CH_4_ selectivity with only 10% reduction in the CO_2_ permeability as compared to corresponding values at 308 K (Figure 1d).

Furthermore, mixed gas permeation tests corroborated the good performance of the hybrid Y‐fum‐**fcu**‐MOF/6FDA‐DAM membrane at subambient temperatures, as shown in Figure 1e. The hybrid membrane has a CO_2_/CH_4_ selectivity as high as ≈130 with CO_2_ permeability of ≈1050 barrers at 233 K and 3.5 bar. Additionally, the membrane performs well under high pressures, even up to 55 bar, at 308 K (Figure 1f). As the pressure increases, CO_2_ permeability and CO_2_/CH_4_ selectivity decreases only gradually indicating minimal plasticization effects. The evaluated membranes sustained their associated performances, thereby affirming that: i) the membrane is mechanically strong and ii) the interface compatibility/interactions between the constituents of the films are sufficient to be maintained and remain operational under wide‐range temperatures. The observed enhancement in the CO_2_/CH_4_ separation performance for the evaluated membranes by fourfold at low temperatures, below ambient temperature, offers great opportunities to combine a membrane system with an appropriate pretreatment to capture condensable components.[[qv: 12a,b,19]]

To gain a better understanding on the fundamentals governing the excellent CO_2_/CH_4_ separation performance at subambient temperature, permeability of CO_2_ and CH_4_ were deconvoluted into diffusivities and sorption coefficients based on sorption–diffusion theory, as shown in **Figure** **2**a,b. As expected, the change in temperature exhibits opposite influences on the gases (CO_2_ and CH_4_) sorption and diffusivity in the membranes. Specifically, gas sorption increases with the decrease of temperature, while gas diffusivity decreases, resulting in minor changes in CO_2_ permeability with temperature variation (Figure 1d) since gas permeability is the product of solubility and diffusivity. Lowering the temperature dramatically increases the CO_2_ sorption in the glassy polymer 6FDA‐DAM, which has excess free‐volume providing abundant sorption sites for CO_2_ at extreme conditions. Interestingly, addition of Y‐fum‐**fcu**‐MOF into 6FDA‐DAM promoted this effect on CO_2_ and CH_4_ solubility when the temperature is lowered. In contrast, lowering the temperature resulted in reduction of CO_2_ and CH_4_ diffusivities due to decreased motions of flexible polymer segments at subambient temperature. The enhanced CO_2_ sorption contributes to the high CO_2_ permeability at subambient temperatures for 6FDA‐DAM and Y‐fum‐**fcu**‐MOF/6FDA‐DAM membranes.

**Figure 2:**
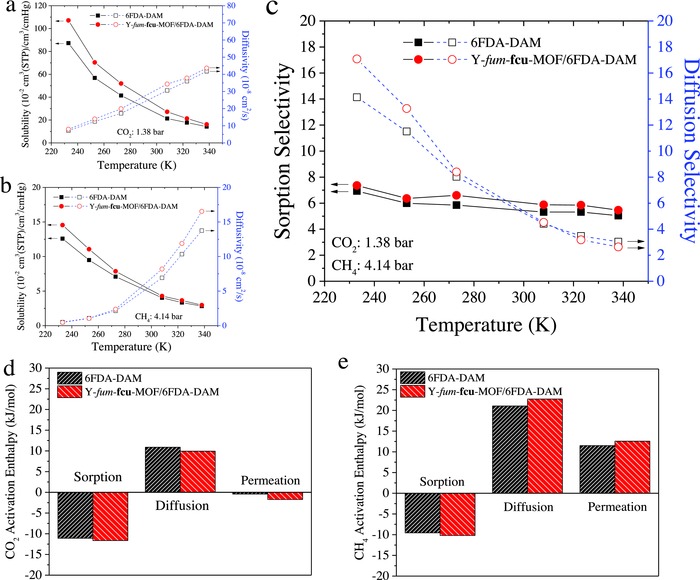
Transport mechanisms of pure 6FDA‐DAM and hybrid 20% Y‐fum‐**fcu**‐MOF/6FDA‐DAM membranes: solubility and diffusivity of a) CO_2_ and b) CH_4_ at 233–338 K. c) Sorption and diffusion based CO_2_/CH_4_ selectivity. Sorption enthalpy, activation enthalpy for diffusion and for permeation of d) CO_2_ and e) CH_4_.

Nevertheless, the sorption factor contributes only in a minor way to the CO_2_/CH_4_ selectivity of the membranes at subambient conditions. Since CH_4_ sorption also increases with decreasing temperature, only small enhancement in CO_2_/CH_4_ sorption selectivity is seen, as shown in Figure 2c. Notably, as the temperature decreases, CO_2_/CH_4_ diffusion selectivity increases rapidly, despite reductions in both CO_2_ and CH_4_ diffusivity (Figure 2a,b). The trend is furtherly strengthened by the addition of Y‐fum‐**fcu**‐MOF, so diffusivity dominates the high CO_2_/CH_4_ selectivity of the membranes at subambient conditions.

Considering diffusion activation enthalpies of CO_2_ and CH_4_ in the membranes provides fundamental understandings on the transport processes. Typically, the activation enthalpy for diffusion is larger in absolute magnitude than the sorption enthalpy, so permeability decreases, and selectivity usually increases with decreasing temperature.^20^ Nonetheless, in this case interaction between the membrane and penetrant promotes high sorption enthalpy, making the sorption enthalpy similar in magnitude or even higher than the activation enthalpy for diffusion. Figure 2d,e shows that the comparable sorption enthalpy and activation enthalpy for diffusion makes CO_2_ permeation high. On the other hand, the much higher activation enthalpy for diffusion than sorption enthalpy induces a high energy barrier for CH_4_ permeation through the membranes. Apparently, the differences between CO_2_ and CH_4_ transport behavior in 6FDA‐DAM membrane can be attributed to: 1) favorable interactions between CO_2_ molecule and the polyimide chains; and 2) lower energy barrier of CO_2_ molecule than that of CH_4_ molecule to execute an effective jump. Furthermore, the addition of Y‐fum‐**fcu**‐MOF increases the diffusive discrimination between these two gas molecules, thereby making the hybrid membrane exhibit a remarkable CO_2_/CH_4_ separation performance at subambient temperatures.

In order to better understand the diffusion process, the CO_2_/CH_4_ diffusion selectivity of the membranes were deconvoluted into energetic selectivity and entropic selectivity, as shown in **Figure** **3**a. The values for pure MOF membrane were also evaluated by back‐calculation using the Maxwell equation.[[qv: 5a]] Detailed descriptions of the deconvolution method can be found in the Supporting Information. Interestingly, unlike carbon molecular sieving (CMS) membranes with slit‐shaped pores showing significant entropic selectivity for CO_2_/CH_4_ separation,^21^ the polymer and hybrid membranes evaluated in this study showed a noteworthy energetic selectivity. This trend is clear from the large differences in diffusion activation enthalpy between CO_2_ and CH_4_ (Figure 2d,e) and low intercepts in Figure 3a. The CO_2_/CH_4_ entropic selectivity of the polymer and hybrid membranes are less than unity, indicating less shape discrimination, versus overall size discrimination as the dominant factor in diffusion selectivity. In fact, the Y*‐fum‐* **fcu**‐MOF with rigid triangle aperture (4.7 Å) has reverse geometrical sieving effect on CO_2_ over CH_4_, though the former has smaller kinetic size (3.3 vs 3.8 Å). Detailed analysis reveals that the significant activation entropic differences of CO_2_ molecule and CH_4_ molecule when passing through the MOF aperture are primarily prompted by their respective distinct molecular shapes. Specifically, CH_4_ molecule is tetrahedral in shape with little difference on three dimensions: *a* = 3.829 Å, *b* = 4.101 Å, *c* = 3.942 Å, while CO_2_ molecule is linear in shape with large difference on its dimensions: *a* = 3.339 Å, *b* = 3.189 Å, *c* = 5.361 Å. Thus the tetrahedral CH_4_ molecule can pass through the aperture without losing any rotational freedom in three axis; however, the long linear CO_2_ molecule has to give up two of its rotational degrees of freedom and some vibrational degrees of freedom to pass through the aperture since its *c*‐dimensional size (5.361 Å) is larger than the aperture size (4.7 Å), as shown in Figure 3b. In this case, traditional kinetic size measured by experiments loses its standard meaning to evaluate the molecular sieving effects. Detailed atomistic understanding of these exceedingly complicated phenomena must await further developments in simulation capabilities.

**Figure 3:**
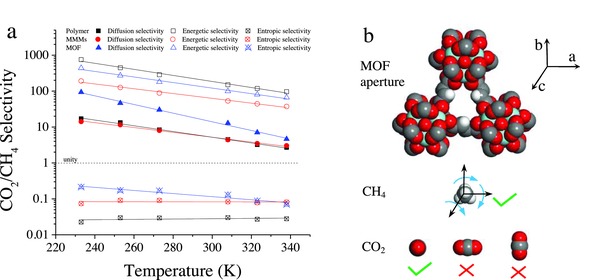
a) CO_2_/CH_4_ diffusion selectivity, energetic selectivity, and entropic selectivity of 6FDA‐DAM, Y‐fum‐**fcu**‐MOF/6FDA‐DAM, and predicted Y‐fum‐**fcu**‐MOF membranes. b) Illustration of Y‐fum‐**fcu**‐MOF aperture as well as CO_2_ and CH_4_ molecules.

With decreasing temperature, CO_2_/CH_4_ energetic selectivity increases, while CO_2_/CH_4_ entropic selectivity remains almost constant, as shown in Figure 3a. This behavior agrees well with fundamental analysis of transition state theory (Supplementary Information). Compared to molecules with higher diffusion activation enthalpy, molecules with lower diffusion activation enthalpy can more easily execute effective jumps. Obviously, by decreasing temperature, this effect due to the diffusion activation enthalpy difference between CO_2_ and CH_4_ is magnified, thereby significantly increasing CO_2_/CH_4_ separation performance of the membranes.

In summary, Y‐fum‐**fcu**‐MOF incorporated 6FDA‐DAM polyimide membrane offers a high CO_2_/CH_4_ separation performance at subambient conditions, overcoming the 2008 Robeson upper bound and the theoretical pure polymer upper bound, even when accounting for the temperature effect. Solution and diffusion play different roles in such a process, dominating the high CO_2_ permeability and high CO_2_/CH_4_ selectivity, respectively. This is attributed to the different physical properties between CO_2_ and CH_4_, e.g., condensability and kinetic diameter, as well as the intrinsic properties of the glassy polymer and the MOF structure, resulting in different levels of activation energies. Moreover, the CO_2_/CH_4_ diffusion selectivity of the membranes is dominated by energetic selectivity. Due to the ultrahigh CO_2_/CH_4_ selectivity and high CO_2_ permeability of the membranes, hybrid natural gas separation process combining membrane with low temperature capture of valuable condensable hydrocarbons may be practical to lower processing cost in comparison with a pure ambient temperature membrane system. Detailed studies involving capital investments evaluation, operation temperature optimization, and robust condition tests are needed in future.

## Experimental Section

*Preparation of Membranes*: The Y‐*fum*‐**fcu**‐MOF crystals were synthesized as reported in the literature.^15^ The as‐synthesized micron‐sized Y‐*fum*‐**fcu**‐MOF crystals were nonideal to form MMMs directly, and thus a mild manual grinding method carrying out at ultralow temperature (e.g., −196 °C using liquid nitrogen) was applied to reduce the crystal sizes as described elsewhere.^16^ The submicron‐sized Y‐*fum*‐**fcu**‐MOF/THF suspension was added to the 6FDA‐DAM/THF solution to form a mixed‐matrix dope, which was then mixed thoroughly on a rolling mixer overnight. Excess solvent (≈60 vol%) in the mixed‐matrix dope was removed by slowly purging dry nitrogen to achieve a higher concentration. Y‐*fum*‐**fcu**‐MOF/6FDA‐DAM mixed matrix dense films with 20 wt% MOF loading were then formed by casting the mixed‐matrix solution. More details can be found in Supplementary Information.

*Gas Sorption and Permeation Tests*: Gas sorption isotherms at pressure up to 14 bar and temperatures ranging from 233 to 338 K were measured using a pressure decay method. The method and apparatus has been described in detail elsewhere.^22^ The gas permeation was conducted in a variable pressure, constant‐volume apparatus. The membrane was housed between an upstream, capable of high‐pressure gas introduction, and a downstream, which was kept under vacuum until experiments were initiated. The permeation temperature for CO_2_ and CH_4_ ranged from 233 to 338 K as described earlier.[[qv: 14c]] A 50/50 (molar) CO_2_/CH_4_ mixture was used for mixed‐gas permeation of Y‐*fum*‐**fcu**‐MOF/6FDA‐DAM membrane. The downstream composition was determined using a gas chromatograph (Varian 450‐GC). The stage cut (the flow rate ratio of permeate to feed) was maintained below 1% to avoid concentration polarization on the upstream side of the permeation cell, keeping the driving force across the membrane constant throughout the course of the experiment.

## Conflict of Interest

The authors declare no conflict of interest.

## Supporting information

Supplementary
