# Supplementary material for: Enhanced CO2/CH4 Separation Performance of a Mixed Matrix Membrane Based on Tailored MOF‐Polymer Formulations
Source: Adv Sci (Weinh). 2018 Aug 2;5(9):1800982. doi: 10.1002/advs.201800982 (PMC6145261; doi:10.1002/advs.201800982)
Supplement: Supplementary file 1 — Supplementary [file ADVS-5-1800982-s001.pdf]

Copyright WILEY-VCH Verlag GmbH & Co. KGaA, 69469 Weinheim, Germany, 2018.

ADVANCED SCIENCE
Open Access

Supporting Information

for *Adv. Sci.*, DOI: 10.1002/advs. 201800982

Enhanced CO2/CH4 Separation Performance of a Mixed Matrix Membrane Based on Tailored MOF-Polymer Formulations

*Yang Liu, Gongping Liu, Chen Zhang, Wulin Qiu, Shouliang Yi, Valeriya Chernikova, Zhijie Chen, Youssef Belmabkhout, Osama Shekhah, Mohamed Eddaoudi, and William Koros**

Copyright WILEY-VCH Verlag GmbH & Co. KGaA, 69469 Weinheim, Germany, 2016.

Supporting Information

Enhanced CO_2/CH_4 Separation Performance of a Mixed Matrix Membrane Based on Tailored MOF-Polymer Formulations

Yang Liu, [a] *Gongping Liu,* [a] *Chen Zhang,* [a] *Wulin Qiu,* [a] *Shouliang Yi,* [a] *Valeriya Chernikova,*[b] *Zhijie Chen,* [b] *Youssef Belmabkhout,* [b] *Osama Shekhah,* [b] *Mohamed Eddaoudi,*[b]*, William Koros* [a,*]

1. Synthesis and cryo-grinding of Y-*fum*-fcu-MOF

Fumaric acid (10.1 mg, 0.087 mmol), $Y(NO_3)_3{\cdot}6H_2O$ (Y = yittrium, 33.4 mg, 0.087 mmol), 2-fluorobenzoic acid (195.0 mg, 1.392 mmol) dimethylformamide (DMF) (2.7 mL), and deionized H_2O (0.7 mL), were combined in a 20 mL scintillation vial, sealed and heated to 115 °C for 72 h and cooled to room temperature. The products were collected and dried at 150 °C under vacuum for 24 h to yield activated sample.

The as-synthesized micron-sized Y-*fum*-**fcu**-MOF crystals were non-ideal to form MMMs directly, thus a mild manual grinding method carrying out at ultra-low temperature (e.g., -196 °C using liquid nitrogen) was applied to reduce the crystals sizes. Specifically, the micron-sized MOF crystals were manually ground in a mortar soaked in liquid nitrogen for 20 min. The ground samples were dispersed in dichloromethane (DCM) to form suspension. After settling 5 min, the submicron-sized MOF crystals suspended in DCM were pipetted out. The resulting DCM suspension containing purified submicron-sized **fcu**-MOF crystals was washed with solvent (THF) for the mixed-matrix membrane preparation.

2. Preparation of membranes

2.1. 6FDA-DAM membranes.

6FDA-DAM were dried in vacuum oven at 110 °C overnight before being dissolved in THF to form 15 wt% polyimide/THF mixtures. The solution was mixed on a rolling mixer overnight to dissolve the polymer. The resulting casting solution was poured onto a glass plate, which was placed in a glove bag pre-saturated with THF vapor for at least 4 h. Pure 6FDA-DAM dense film was formed on a glass plate by simple casting the desired thickness (typically 75 μm) using a draw knife with appropriate specific clearance. The films were left in the glove bag overnight to allow the THF solvent to evaporate slowly and then dried in a vacuum oven at 200 °C for 20 hrs.

2.2. Y-*fum*-**fcu**-MOF/6FDA-DAM mixed-matrix membranes.

The submicron-sized Y-*fum*-**fcu**-MOF/THF suspension was added to the 6FDA-DAM/THF solution to form a mixed-matrix dope, which was then mixed thoroughly on a rolling mixer overnight. Excess solvent (~60 vol%) in the mixed-matrix dope was removed by slowly purging dry nitrogen to achieve a higher concentration. Y-*fum*-**fcu**-MOF/6FDA-DAM mixed matrix dense films with 20 wt% MOF loading were then formed by casting the mixed-matrix solution in the same condition as for the pure 6FDA-DAM dense films.

3. Characterization of Materials

The dense films for scanning electron microscope (SEM, Hitachi, SU8010) test were prepared by first soaking films in hexane and then cryogenically fracturing in liquid nitrogen to preserve their microstructures. Wide-angle X-ray diffraction (WAXD) was measured on a Panalytical Empyrean diffractometer operating with a Cu Kα radiation at a wavelength of 1.54 Å, in a 2θ range of 5 - 50°. Thermogravimetric analysis (TGA) and derivative weight data were recorded on a TA Q-500 analyzer at a heating rate of 10 °C/min under a nitrogen atmosphere, using ~10 mg sample. The MOF loading in mixed-matrix dense films was determined by a TGA method.

4. Pure Gas Sorption Tests

Gas sorption isotherms at pressure up to 14 bar and temperatures ranging from 233 K to 308 K were measured using a pressure decay method. A BTZ-475 benchtop temperature chamber (ESPEC North Amercia, INC., Hudsonville, MI), which accurately controlled temperature within the range of 203 K to 453 K with 0.5 K fluctuation, was used for sorption and permeation measurement.

All samples were activated at 150 °C under vacuum for 24 h and then loaded into cell (B) and degassed for overnight. Some sorption gas was first introduced into the reservoir (A), then the connecting valve was quickly open for 1 second and closed. The pressure signal in both volume (A) and volume (B) were recorded continuously. The gas adsorption concentration in films or MOFs was calculated through a mole balance: adsorbed amount equals to decreased amount in reservoir volume (V_A) minus increased amount of gas in cell volume (V_B), as below:

$$C = \frac{\frac{V_A}{R\cdot T}\left(\frac{p_{A,i}}{z_{A,i}}-\frac{p_{A,f}}{z_{A,f}}\right)-\frac{V_B}{R\cdot T}\left(\frac{p_{B,f}}{z_{B,f}}-\frac{p_{B,i}}{z_{B,i}}\right)}{V_{sample}} \tag{S1}$$

Gas adsorption amount in Y-fcu-MOFs and dense films was calculated from the pressure change before and after sorption with a small experimental error of less than ± 5 % expected from the accurate pressure measurement and volume calibration.

5. Gas permeation Tests

The gas permeation was conducted in a variable pressure, constant-volume apparatus. The membrane was housed between an upstream, capable of high-pressure gas introduction, and a downstream, which is kept under vacuum until experiments were initiated. The permeation temperature for CO_2 and CH_4 ranges from 233 K to 338 K.

A 50/50 (molar) CO_2/CH_4 mixture was used for mixed-gas permeation of Y-*fum*-**fcu**-MOF/6FDA-DAM membrane. The downstream composition was determined using a gas chromatograph (Varian 450-GC). The stage cut (the flow rate ratio of permeate to feed) was maintained below 1% to avoid concentration polarization on the upstream side of the permeation cell, keeping the driving force across the membrane constant throughout the course of the experiment.

6. Permeability, Solubility, Diffusivity and Energetic Factors

Permeability and selectivity were used to characterize the membrane separation performance. The permeability, P_i, describes the intrinsic gas separation productivity of a dense film membrane and is defined by the flux of penetrant *i*, n_i, normalized by the membrane thickness, *l*, and the partial pressure or fugacity difference, Δf_i, across the membrane, viz.,

$$P_i = \frac{n_i \cdot l}{\Delta f_i} \quad \text{(S2)}$$

In order to estimate pure gas permeability, the slope of the permeate pressure vs. time (*df/dt*); membrane thickness (*l*); downstream volume (*V*); operating temperature (*T*); and transmembrane pressure or fugacity difference (Δf) were used with Equation (3):

$$P = \frac{\frac{dp}{dt} \cdot l \cdot V}{A \cdot T \cdot \Delta f} \quad \text{(S3)}$$

The mixed gas permeability coefficient of component *i* (P_i) is calculated using its mole fraction in the permeate (x_i) and the transmembrane fugacity difference (Δf_i):

$$P_i = \frac{\frac{dp}{dt} \cdot x_i \cdot l \cdot V}{A \cdot T \cdot \Delta f_i} \quad \text{(S4)}$$

The fugacity coefficients are calculated using Peng-Robinson equation-of-state and the SUPERTRAPP program developed by NIST. The CO_2/CH_4 selectivity, α_{ij}, is determined by the ratio of the fast gas (*i*, CO_2) permeability to the slow gas (*j*, CH_4):

$$\alpha_{ij} = \frac{P_i}{P_j} \quad \text{(S5)}$$

Permeability can also be expressed as the product of the average effective diffusion coefficient (*Di*) and sorption coefficient ($\tilde{S}_i$) of a given gas *i* within the membrane:

$$P_i = D_i \cdot \tilde{S}_i \quad \text{(S6)}$$

The sorption coefficient represents the thermodynamic contribution to transport, which can be measured independently by pressure-decay sorption. The sorption coefficient can be expressed as:

$$\tilde{S}_i = \frac{c_i}{f_i} \tag{S7}$$

where c_i is the concentration of a gas adsorbed in the sample, and f_i is the corresponding upstream fugacity driving force of component *i*. In this work, the adsorbed gas concentration in films was described by the dual-mode sorption model, which is given as:

$$c_i = c_{D,i} + c_{H,i} = k_{D,i} \cdot f_i + \frac{C'_{H,i} \cdot b_i \cdot f_i}{1 + b_i \cdot f_i} \tag{S8}$$

where $c_{D,i}$ is the Henry's law or dissolved mode penetrant concentration, $c_{H,i}$ is the penetrant concentration in the Langmuir mode or hole-filling sorption mode. The $k_{D,i}$ is the Henry's law sorption coefficient reflecting properties of polymer matrix. On the other hand, $C'_{H,i}$ is the Langmuir capacity constant, and b_i is the Langmuir affinity constant, which reflect the average of these parameters contributed by the glassy polymer and MOF.

The effective diffusion coefficient (*Di*) in the membrane was calculated from the independently measured permeability (P) and sorption coefficient ($\tilde{S}_i$):

$$\widetilde{D}_i = \frac{P_i}{\tilde{S}_i} \tag{S9}$$

The temperature dependence of sorption can be described by the van’t Hoff equation:

$$\tilde{S} = \tilde{S}_0 exp\left(\frac{-\Delta H_S}{RT}\right) \tag{S10}$$

where $\tilde{S}_0$ is the pre-exponential factor, ΔH_S is the sorption enthalpy. The temperature dependence of the diffusion coefficient follows an Arrhenius relationship:

$$D = D_0 \exp\left(\frac{-E_D}{RT}\right) \tag{S11}$$

where D_0 is the pre-exponential factor, E_D is the activation energy for diffusion. The E_D parameter represents the energy required for a penetrant to jump between adsorption sites within the material, which is primarily dependent on penetrant size and shape. The temperature dependence of permeability also follows an Arrhenius relationship:

$$P = P_0 exp\left(\frac{-E_P}{RT}\right) \tag{S12}$$

where P_0 is the pre-exponential factor, and E_P is the effective activation energy of permeation, E_P, E_D and ΔH_S have the following relationship:

$$E_P = E_D + \Delta H_S \tag{S13}$$

Expressing the diffusion temperature dependence in terms of the activation enthalpy, the diffusion selectivity can also be expressed as the product of energetic selectivity and entropic selectivity based on transition state theory by replacing the activation energy approximately with the activation enthalpy:

$$\frac{D_A}{D_B} = \frac{\lambda_A}{\lambda_B} \underbrace{\left[exp\left(\frac{S_{DA}^{\ddagger} - S_{DB}^{\ddagger}}{R}\right)\right]}_{\text{Entropic selectivity}} \underbrace{\left[exp\left(-\frac{H_{DA}^{\ddagger} - H_{DB}^{\ddagger}}{RT}\right)\right]}_{\text{Energetic selectivity}} \tag{S14}$$

where A and B represent different penetrants. Since $H_{Di}^{\ddagger}$ = E_{Di} + RT, we can write $H_{DA}^{\ddagger} - H_{DB}^{\ddagger} = E_{DA}^{\ddagger} - E_{DB}^{\ddagger} = \Delta E_{DAB}$. Energetic selectivity reflects the difference in the diffusion activation energies of the two penetrants, while entropic selectivity reflects the difference in the diffusion activation entropies of the two penetrants. This entropic configurational control can be engineered by controlling differences in rotational and internal vibrational degrees of freedom between the diffusing components. Although approximate for hybrid materials with different jump lengths in the continuous and dispersed phases, in the absence of perfect molecular sieving, both penetrants follow the same jump lengths in the continuous and dispersed phases. On this basis, the jump length ratios can be cancelled to arrive at a simplified form of Equation S14, which allows analysis on energetic vs entropic selectivity factors in the MOF MMMs materials,

$$\frac{D_A}{D_B} = \left[exp\left(\frac{S_{DA}^{\ddagger} - S_{DB}^{\ddagger}}{R}\right)\right]\left[exp\left(-\frac{\Delta E_{DAB}}{RT}\right)\right] \tag{S15}$$

7. Analysis and validation of diffusion selectivity based on transition state theory

As shown above, neglecting any small differences in jump lengths and difference between average activation energy and average activation enthalpy in the material, the diffusion selectivity can be expressed as the product of energetic selectivity and entropic selectivity:

$$\frac{D_A}{D_B} = \left[exp\left(\frac{S^{\ddagger}_{DA} - S^{\ddagger}_{DB}}{R}\right)\right]\left[exp\left(-\frac{\Delta E_{DAB}}{RT}\right)\right] \quad \text{(S15)}$$

Furthermore, Euqation S15 can also be expressed as:

$$\ln\left(\frac{D_A}{D_B}\right) = -\frac{\Delta E_{DAB}}{RT} + \frac{S^{\ddagger}_{DA} - S^{\ddagger}_{DB}}{R} \quad \text{(S16)}$$

$$\ln\left(\frac{D_A}{D_B}\right) = (lnD_A - lnD_B) = -\frac{\Delta E_{DAB}}{R} \times \frac{1}{T} + \frac{S^{\ddagger}_{DA} - S^{\ddagger}_{DB}}{R} \quad \text{(S17)}$$

By assuming E_{DA}, E_{DB}, S_{DA} and S_{DB} are constants, $\ln\left(\frac{D_A}{D_B}\right)$ and $\frac{1}{T}$ (K^{-1}) have a linear relationship (Figure S1):

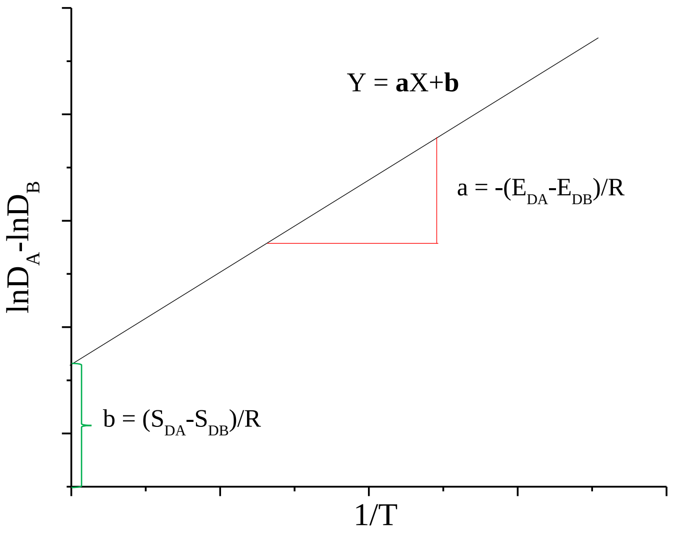

Figure S1. Relationship between (lnD_A-lnD_B) and 1/T.

Obviously, the energetic selectivity $\left[exp\left(-\frac{\Delta E_{DAB}}{R} \times \frac{1}{T}\right)\right]$ is slightly temperature dependent, while entropic selectivity $\left[exp\left(\frac{S^{\ddagger}_{DA} - S^{\ddagger}_{DB}}{R}\right)\right]$ should not affected by temperature.

The total diffusion selectivity $\frac{D_A}{D_B}$, as a production of energetic selectivity and entropic selectivity, is also slightly temperature dependent.

The differential values of energetic and entropic energies between CO_2 and CH_4 derived from Equation S17 matches well with those of traditional method. Taking CO_2 and CH_4 diffusion in 6FDA-DAM as example, ($\ln D_{CO2}$-$\ln D_{CH4}$) and 1/T have the following relationship:

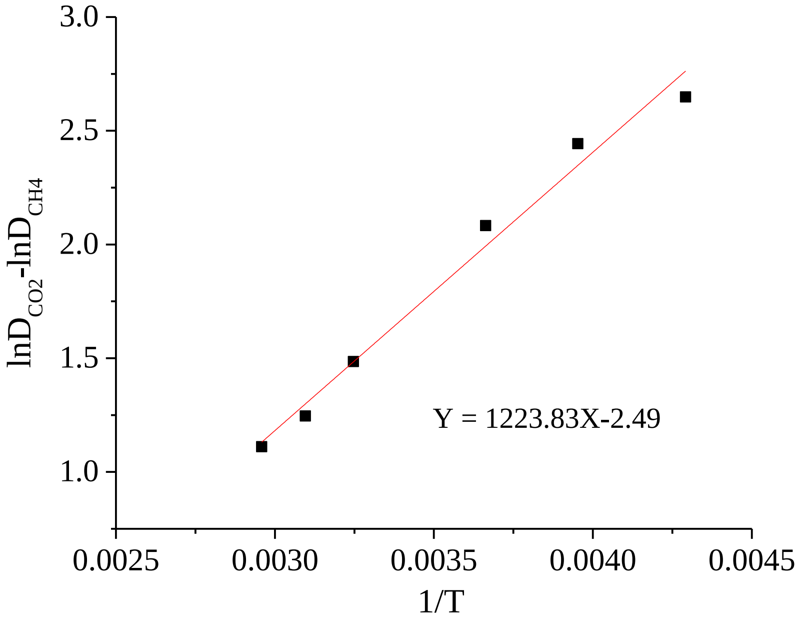

Figure S2. Relationship between ($\ln D_{CO2}$-$\ln D_{CH4}$) and 1/T for 6FDA-DAM membrane. The partial pressures are 20 psi and 60 psi for CO_2 and CH_4, respectively.

Here,

$$-\frac{\Delta E_{DAB}}{RT} = 1223.83 \quad \text{(S18)}$$

In Figure 2 in the manuscript, the calculated diffusion activation energy for CO_2 and CH_4 are 10.88 kJ/mol and 21.06 kJ/mol, respectively. Thus we can get

$$-\frac{E_{DA}-E_{DB}}{R} = -\frac{10.88-21.06}{8.314} \times 1000 = 1224.44 \approx 1223.83 \quad \text{(S19)}$$

which indicates the good match of our results using different methods.

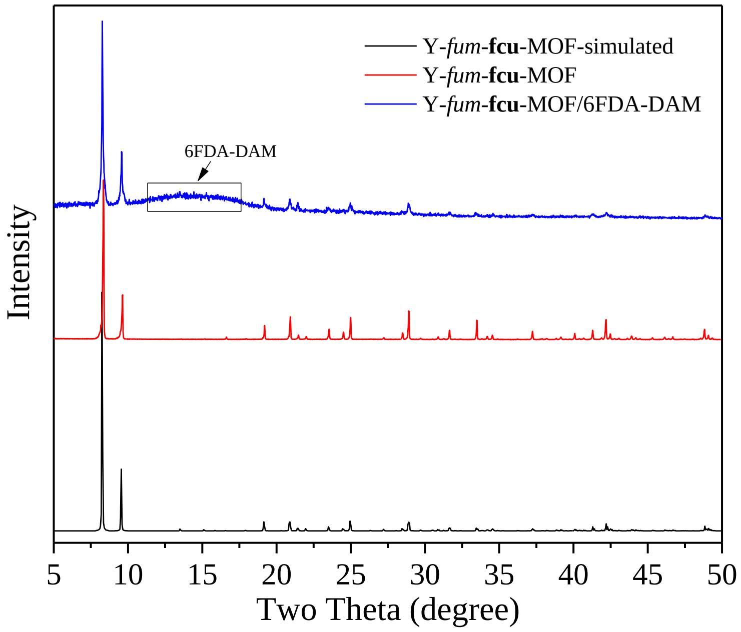

Figure S3. simulated and experimental XRD of Y-*fum*-**fcu**-MOF and hybrid 20% Y-*fum*-**fcu**-MOF/6FDA-DAM membrane.

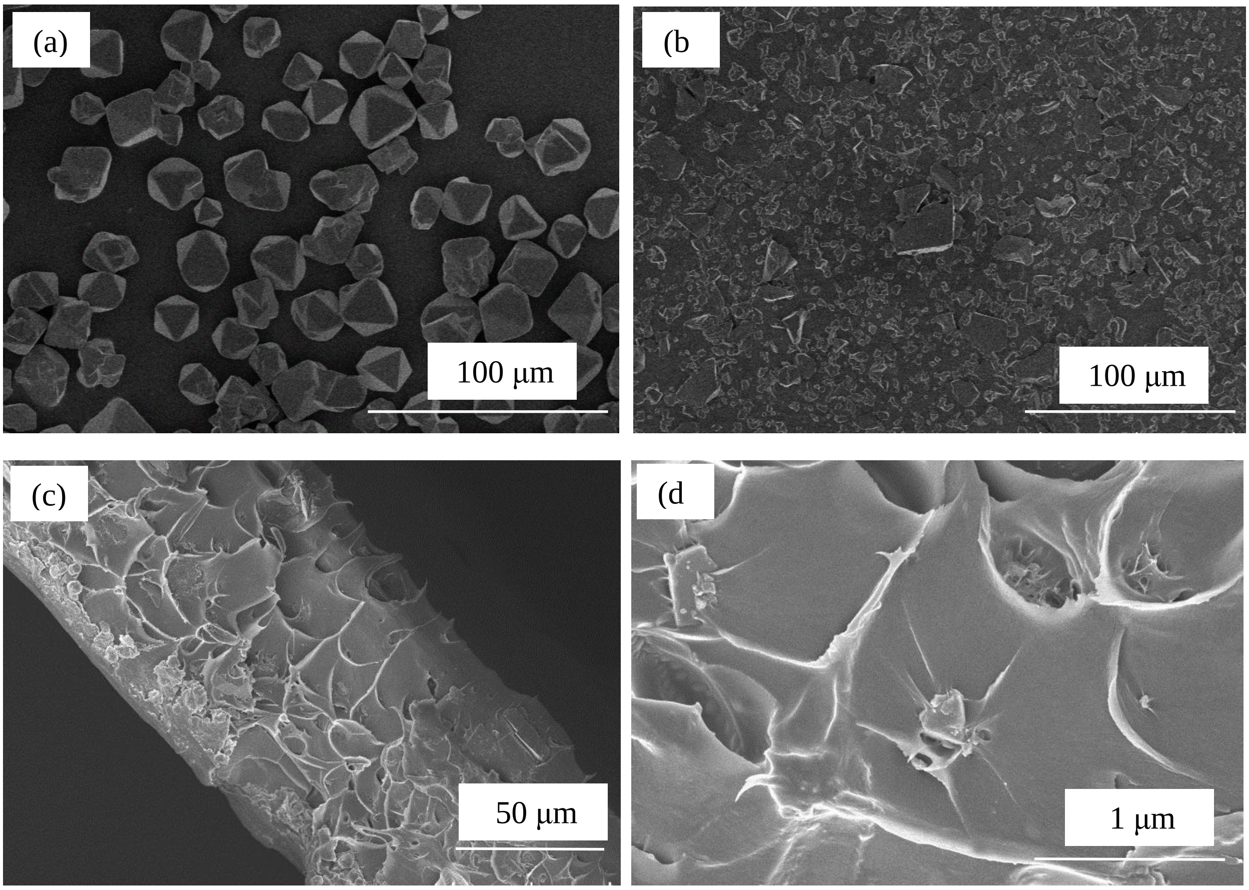

Figure S4. SEM images of (a) pristine Y-fum-**fcu**-MOF crystals; (b) cryo-ground Y-fum-**fcu**-MOF crystals; (c) Y-fum-**fcu**-MOF/6FDA-DAM membrane; (d) interface between MOF and polymer.

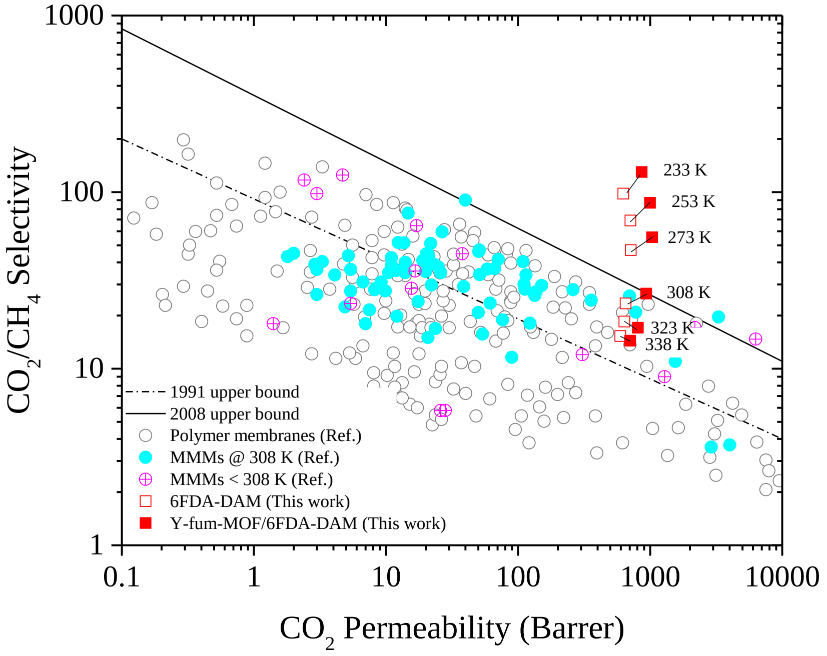

Figure S5. CO_2/CH_4 separation performance of the membranes fabricated in this work in comparison with reported polymer membranes and mixed matrix membranes as well as upper bounds.

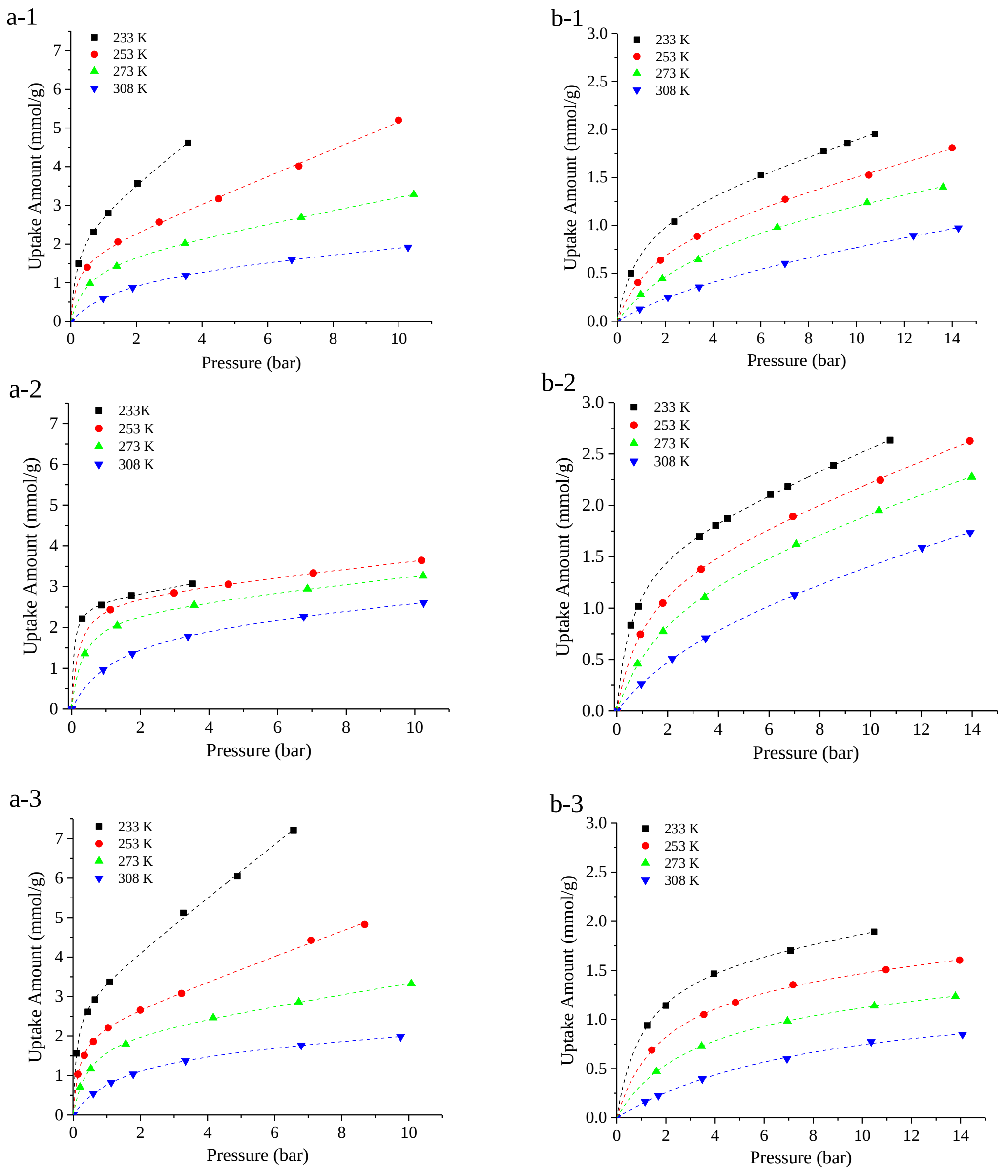

Figure S6. Equilibrium sorption isotherms of CO_2 (a) and CH_4 (b) on 6FDA-DAM (1), Y-*fum*-**fcu**-MOF (2) and Y-*fum*-**fcu**-MOF/6FDA-DAM (3) at 233 K - 338 K.
